# Supplementary material for: Improving qubit coherence using closed-loop feedback
Source: Nat Commun. 2022 Apr 11;13:1932. doi: 10.1038/s41467-022-29287-4 (PMC9001732; doi:10.1038/s41467-022-29287-4)
Supplement: Supplementary file 1 — Supplementary Information [file 41467_2022_29287_MOESM1_ESM.pdf]

Supplementary information: Improving qubit coherence using closed-loop feedback

Antti Vepsäläinen,[1,*] Roni Winik,[1] Amir H. Karamlou,[2,1] Jochen Braumüller,[1] Agustin Di Paolo,[1] Youngkyu Sung,[2] Bharath Kannan,[2] Morten Kjaergaard,[1,3] David K. Kim,[4] Alexander J. Melville,[4] Bethany M. Niedzielski,[4] Jonilyn L. Yoder,[4] Simon Gustavsson,[1] and William D. Oliver[2,4]

[1]*Research Laboratory of Electronics, Massachusetts Institute of Technology*
[2]*Department of Electrical Engineering and Computer Science, Massachusetts Institute of Technology*
[3]*Center for Quantum Devices, University of Copenhagen*
[4]*MIT Lincoln Laboratory*
(Dated: February 20, 2022)

* apvepsala@gmail.com

A. Experimental setup and the hardware implementation

Fig. S1 shows the hardware setup used in the experiment. The model numbers of the used instruments are provided in Table I. The qubit XY control and readout pulses are created by an arbitrary waveform generator (AWG) (Keysight M3202A). The in-phase and quadrature pulses are up-converted to the qubit and the resonator frequencies using two RF sources (R&S SGMA SGS100A), which have a built-in IQ-mixer used for single-sideband modulation. The signals are attenuated to mitigate thermal noise from room-temperature, and sent to the qubit installed in a Leiden Cryogenics dilution refrigerator. After interacting with the readout resonator, the readout signal is amplified by a traveling wave parametric amplifier (TWPA), which is driven by a pump tone (Keysight E8267D). The TWPA is followed by a chain of conventional amplifiers and the signal is digitized at room-temperature using a digitizer with an on-board FPGA programmed to discriminate the qubit states (Keysight M3102A).

TABLE I. The used control equipment.

instrument	vendor	model
Electronics chassis	Keysight	M9019A
XY control LO source	R&S	SGS100A
XY control AWG	Keysight	M3202A
flux control AWG	Keysight	M3202A
DC flux bias	Yokogawa	GS200
readout LO source	R&S	SGS100A
readout AWG	Keysight	M3202A
readout digitizer	Keysight	M3102A
TWPA pump	Keysight	E8267D
Control software	Keysight	Labber

The qubit flux bias is controlled by two channels of another AWG (Keysight M3202A). The first channel is used to set the operating point for the qubit, whereas the second channel is controlled by the AWG's on-board FPGA to set up the feedback loop. The feedback channel is heavily attenuated in order to use the full voltage bandwidth of the AWG to reduce the discretization noise.

The feedback loop is formed together by the readout digitizer and the qubit flux control AWG. The on-board FPGA of the digitizer is used to digitally demodulate and integrate the readout signal and then use a threshold discriminator to assign the qubit state q_i to either 0 or 1, see Fig. S2 for the block diagram of the FPGA operation. The discriminated states are transferred to the AWG for the calculation of the feedback signal.

First we perform the virtual qubit state reset by flipping the discriminated state q_i if the previous discriminated state was excited state. This is equivalent to $q_i \rightarrow \mathrm{XOR}(q_i, q_{i-1})$. In the next step, the q_i are used to calculate the estimate for the qubit state $s_i = N\hat{p}_1 = \sum_{j=i-N+1}^{i} q_j = s_{i-1} - q_{i-N+1} + q_i$. The value of the buffer sum s is used as an index for a lookup table which contains the binary representation of the voltages corresponding to each of the possible $N+1$ values for s_i. In the last step, the voltage values are fed to an accumulator, which value is updated every N_S steps. We set $N_\mathrm{S} = N$ for the measurement of the qubit frequency fluctuation spectral density, and $N_\mathrm{S} = N+1$ for the interleaved operation.

B. Qubit frequency estimation

The qubit frequency can be estimated using a simple version of the single qubit phase estimation algorithm where the qubit is first prepared in a superposition state with a $\pi/2$ rotation around Y axis followed by free evolution. During the free evolution, the qubit wavefunction accumulates a phase (in the frame rotating with the drive) $\phi(t) = \int_0^t 2\pi\delta_\mathrm{q}(t)\mathrm{d}t$, where $\delta_\mathrm{q}(t) = f_\mathrm{d} - f_\mathrm{q}(t)$ and f_d and $f_\mathrm{q}(t)$ are the drive frequency and the qubit frequency. The accumulated phase can be estimated by measuring either $\langle X\rangle$ or $\langle Y\rangle$. During the experiment, the qubit state evolves as

$$|\psi\rangle = \exp\left(-\frac{i\pi}{4}M\right)\exp\left(-i\frac{\pi}{2}\phi(\tau)Z\right)\exp\left(-\frac{i\pi}{4}Y\right)|\psi_0\rangle, \tag{S1}$$

where $|\psi_0\rangle$ is the initial state of the qubit, τ is the free-evolution time, M is the measurement operator, and $\phi(\tau) = 2\pi\tau\delta_\mathrm{q}$ if the detuning δ_q is assumed to be constant during the experiment. The probability of measuring the excited state is given by

$$p_1 = \frac{1-\langle\psi|Z|\psi\rangle}{2} = \frac{1}{2} + \frac{1}{2}\cos(2\pi\delta_\mathrm{q}\tau - \phi_\mathrm{m}), \tag{S2}$$

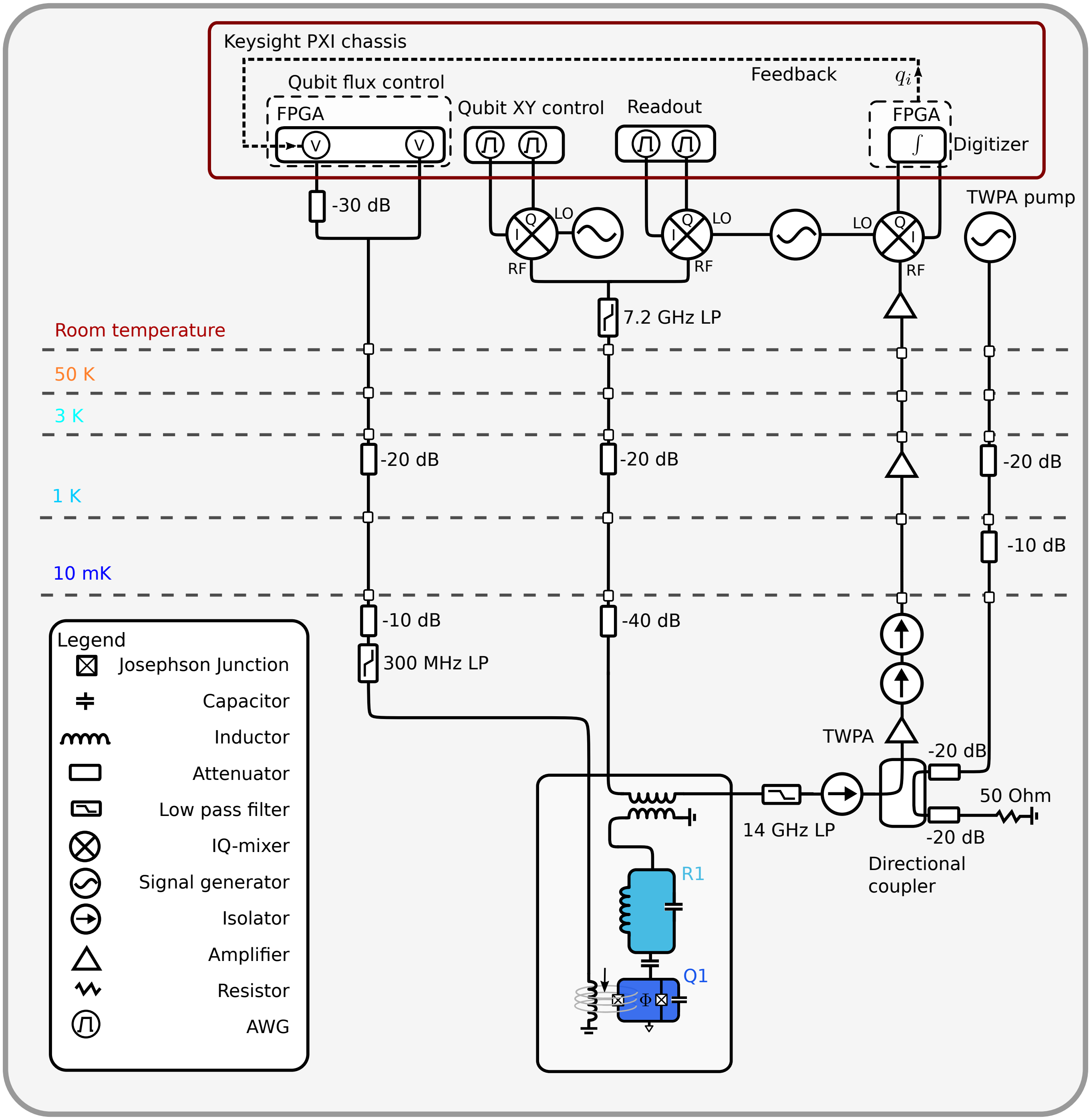

FIG. S1. The experimental setup.

where ϕ_m is a phase factor that depends on the measurement operator,

$$\phi_\mathrm{m} = \begin{cases} 0, & \text{if } M = X, \\ \pi/2, & \text{if } M = Y. \end{cases} \tag{S3}$$

Solving for δ_q yields

$$\delta_\mathrm{q} = \frac{\pm \arccos(2p_1 - 1) + \phi_\mathrm{m} + 2\pi k}{2\pi\tau}. \tag{S4}$$

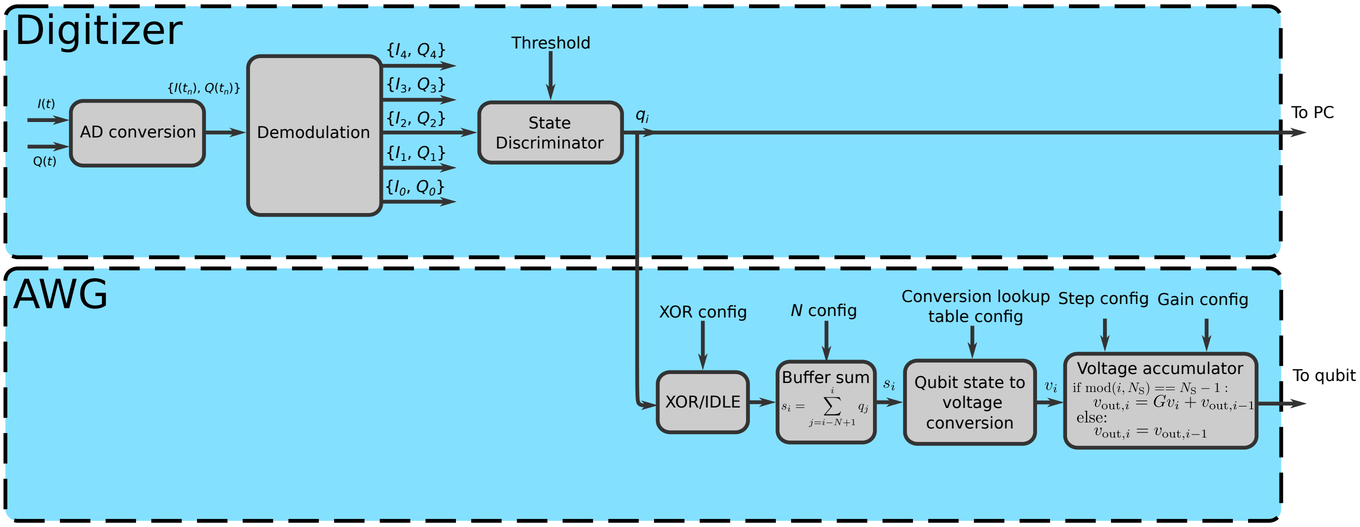

FIG. S2. The block diagram of the two FPGAs performing the feedback. The FGPA in the digitizer discriminates the state, and forwards the information to the FPGA which controls the AWG.

By choosing $k = 0$ and the negative branch δ_q is uniquely defined in the range $\delta_\mathrm{q} = [\frac{\phi_\mathrm{m}-\pi}{2\pi\tau}, \frac{\phi_\mathrm{m}}{2\pi\tau}]$, which is symmetric around zero for $\phi_\mathrm{m} = \frac{\pi}{2}$.

The qubit excited state probability $\hat{p}_1$ can be estimated from N measurements of the qubit state q_i through $\hat{p}_1 = \frac{1}{N}\sum_{i=0}^{N} q_i$, which then gives an estimate of the qubit frequency $\hat{\delta}_\mathrm{q}$. However, this frequency estimate is noisy due to finite number of samples used to estimate the excited state probability. The impact of the noise can be estimated by noting that $\hat{p}_1$ is binomially distributed, and its standard deviation is

$$\delta\hat{p}_1 = \sqrt{\frac{p_1(1-p_1)}{N}} = \frac{1}{2\sqrt{N}}, \text{ for } p_1 = 1/2. \tag{S5}$$

The error in the frequency estimate is then given by

$$\delta\hat{\delta}_\mathrm{q} = \left|\frac{\partial\delta_\mathrm{q}}{\partial p_1}\delta\hat{p}_1\right| \approx \left|\frac{\partial}{\partial p_1}\frac{\frac{\pi}{2}+\phi_\mathrm{m}+2p_1-1}{2\pi\tau}\delta\hat{p}_1\right| = \frac{1}{2\pi\tau\sqrt{N}}, \tag{S6}$$

where δ_q has been expanded to the first order in p_1. This implies that the estimation noise can be reduced either by increasing τ or the number of measurements used in the estimation. However, the bandwidth of the estimation algorithm is limited by the requirement that each frequency has to be uniquely given by p_1. This is true if $|\delta_\mathrm{q}\tau| \leq \frac{1}{4}$. As a result, there is a trade-off between the estimation bandwidth and sensitivity. Decoherence further reduces the sensitivity, and the optimum is obtained at $\tau = T_2$ [37]. In practice, τ often needs to be shorter to satisfy the bandwidth requirement. Using a larger N increases the total time taken by the frequency estimation, which reduces the repetition rate of the feedback algorithm.

C. Circuit analysis

Here we theoretically analyze the implemented feedback circuit and assess its performance. We start by writing the block diagram for the circuit, shown in S3a. In the diagram and the following calculation, the quantities are sampled at times $t = nT_\mathrm{N} = nNT$, where T is the total duration of a Ramsey experiment and N is the number of Ramsey experiments used to estimate the qubit frequency. The target qubit frequency is controlled by $d[n] = f_\mathrm{d}$ which we keep fixed at all times. At every cycle of the feedback, the target frequency $d[n]$ is compared to the estimated qubit frequency $f[n]$, resulting in the error signal $e[n] = d[n] - (f[n] + v[n])$, where $v[n]$ is the sampling noise. In the experiment, the error signal is given by $e[n] = \hat{\delta}_\mathrm{q}[n]$, and the sampling noise $v[n] = \delta\hat{\delta}_\mathrm{q}[n]$ is defined in Eq. (S6). The error signal is multiplied by a controllable gain G, and is fed to an accumulator outputting the feedback control signal $p[n-1]$ — delayed by one time step — which then adjusts the qubit frequency.

The sampled qubit frequency $f[n]$ is estimated from the real qubit frequency $f_\mathrm{q}(t)$ using N Ramsey measurements as described in the main text. Here, we approximate the sampled frequency as the average qubit frequency during

the sampling

$$f[n] \approx \frac{1}{N\tau} \sum_{i=0}^{N-1} \int_{nT_{\mathrm{N}}+iT}^{nT_{\mathrm{N}}+iT+\tau} f_{\mathrm{q}}(t)\mathrm{d}t. \tag{S7}$$

The qubit frequency is determined by its intrinsic fluctuating value $\tilde{f}_{\mathrm{q}}$ which is acted on by the feedback signal $p[n-1]$ so that $f_{\mathrm{q}}(t) = \tilde{f}_{\mathrm{q}}(t) + p[n-1]$. Substituting to Eq. (S7) yields

$$f[n] \approx p[n-1] + \frac{1}{N\tau} \sum_{i=0}^{N-1} \int_{nT_{\mathrm{N}}+iT}^{nT_{\mathrm{N}}+iT+\tau} \tilde{f}_{\mathrm{q}}(t)\mathrm{d}t \equiv p[n-1] + \tilde{f}[n], \tag{S8}$$

forming the feedback loop.

To analyze the impact of the feedback on the signal and the sampling noise, we start by solving the transfer function of the feedback signal $p[n]$ from

$$\begin{aligned} & e[n] = d[n] - v[n] - \tilde{f}[n] - p[n-1], \\ & p[n] = p[n-1] + y[n], \\ & y[n] = Ge[n], \\ \implies & p[n] = p[n-1] - G(-d[n] + v[n] + \tilde{f}[n] + p[n-1]). \end{aligned} \tag{S9}$$

Applying the z-transform yields

$$\begin{aligned} P(z) &= z^{-1}P(z) - G(-D(z) + V(z) + \tilde{F}(z) + z^{-1}P(z)), \\ P(z) &= \frac{G(D(z) - V(z) - \tilde{F}(z))}{1 - z^{-1} + z^{-1}G}, \end{aligned} \tag{S10}$$

resulting in the transfer function

$$X_{\mathrm{p}}(z) = P(z)/(D(z) - V(z) - \tilde{F}(z)) = \frac{G}{1 - z^{-1} + z^{-1}G}. \tag{S11}$$

To get the frequency response $H_{\mathrm{p}}(f)$ from the transfer function in Eq. (S11), we substitute $z = \mathrm{e}^{i2\pi fNT}$ so that $H_{\mathrm{p}}(f) = X_{\mathrm{p}}(\mathrm{e}^{i2\pi fNT})$, which is shown in Fig. S3b for $G = 0.35$. The power spectral density of the feedback signal as a response to the frequency fluctuations of the system is given by the squared magnitude of the frequency response [38]

$$S_{\mathrm{pp}}(f) = |H_{\mathrm{p}}(f)|^2 \, S_{\mathrm{ss}}(f), \tag{S12}$$

where $S_{\mathrm{ss}}(f)$ is the combined power spectral density of the target signal $d[n]$, the sampled intrinsic qubit frequency $\tilde{f}[n]$ and the sampling noise $v[n]$, $S_{\mathrm{ss}}(f) = S_{\mathrm{dd}}(f) + S_{\tilde{\mathrm{f}}\tilde{\mathrm{f}}}(f) + S_{\mathrm{vv}}(f)$. We assume that there is only a minimal amount of noise in frequency of the microwave source which sets the target frequency, implying that we can set $S_{\mathrm{dd}} \approx 0$. To maximize the efficiency of the feedback, we limit the bandwidth of the frequency response to the frequencies where the spectral density of the qubit frequency fluctuations is higher than the sampling noise. Another option would be to increase number of phase sampling experiments N per frequency estimate, which reduces the sampling noise power at the cost of a longer total time used for feedback.

To better understand the experimentally measured spectrum in Fig. 1d of the main text, we next calculate the frequency response of the error signal $e[n]$ from Eqs. (S9). Applying the z-transform and solving for $E(z)$ yields

$$\begin{aligned} & E(z) = D(z) - V(z) - \tilde{F}(z) - z^{-1}P(z), \\ & P(z) = z^{-1}P(z) + GE(z), \\ \implies & E(z) = \frac{1 - z^{-1}}{1 - z^{-1} + Gz^{-1}}(D(z) - V(z) - \tilde{F}(z)), \\ \implies & X_{\mathrm{e}}(z) = \frac{1 - z^{-1}}{1 - z^{-1} + Gz^{-1}}. \end{aligned} \tag{S13}$$

The frequency response $H_{\mathrm{e}}(f) = X_{\mathrm{e}}(\mathrm{e}^{i2\pi fNT})$ is plotted in Fig. S3b. Without the feedback, the expected spectral density of the error signal is $S_{\mathrm{ee}}^{\mathrm{nofeedback}}(f) = S_{\tilde{\mathrm{f}}\tilde{\mathrm{f}}}(f) + S_{\mathrm{vv}}(f)$. When the feedback is turned on, the response is

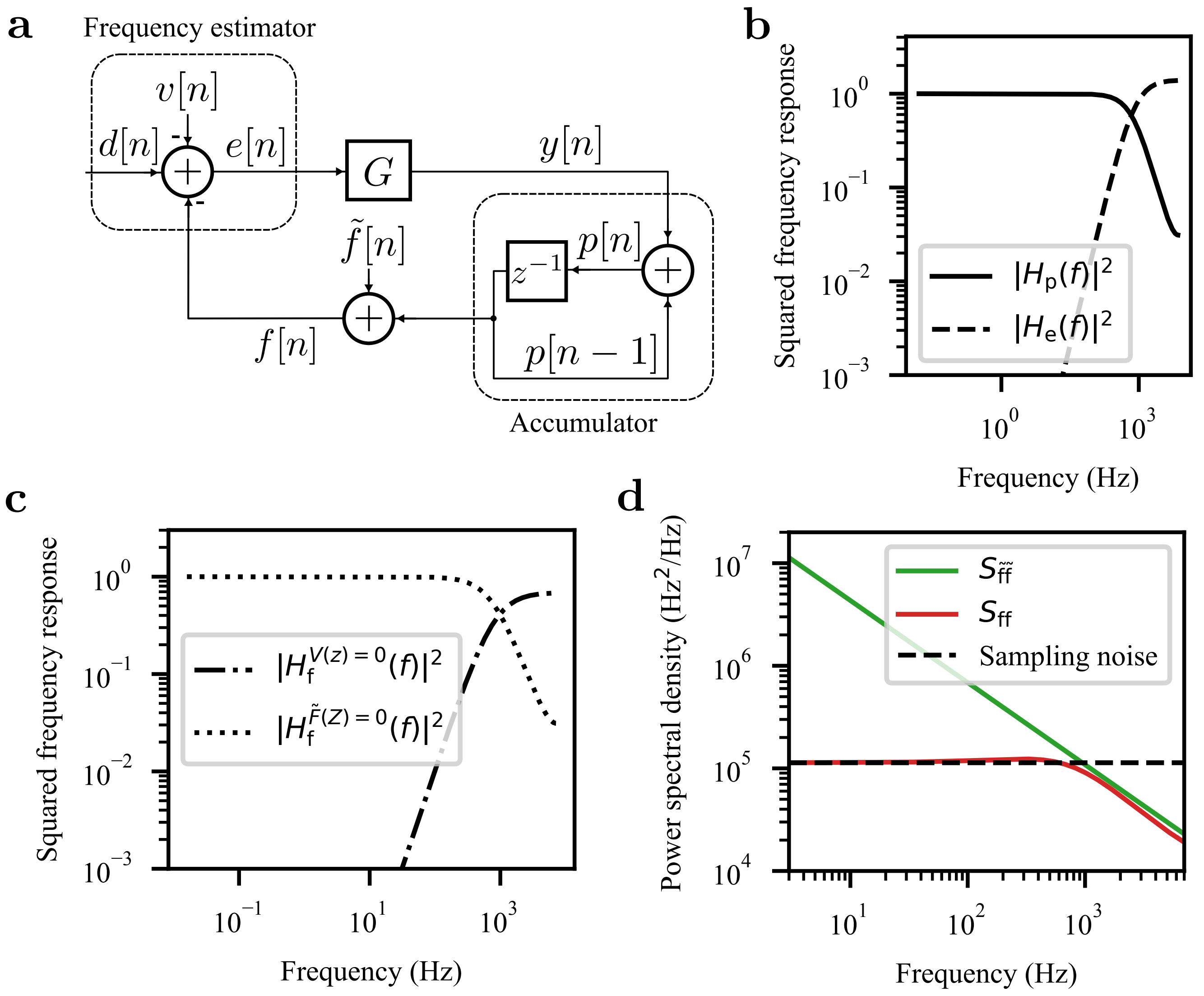

FIG. S3. a) The diagram of the feedback circuit. b) The squared magnitude of the frequency responses for the control and error signals $p[n]$ and $e[n]$. c) The squared magnitude of the frequency response for the qubit frequency during the feedback. The dashed line shows the frequency response of the qubit to the sampling noise, and the dash-dotted line shows the frequency response to the fluctuations in the intrinsic qubit frequency. d) The power spectral density of the qubit frequency fluctuation with the feedback activated (red) calculated using the frequency responses shown in c) and the experimentally measured spectral density of the intrinsic qubit frequency fluctuation (green). The dashed line shows the sampling noise.

$S_{\mathrm{ee}}^{\mathrm{feedback}} = (S_{\tilde{\mathrm{f}}\tilde{\mathrm{f}}}(f) + S_{\mathrm{vv}}(f))|H_{\mathrm{e}}(f)|^2$ instead. At lower frequencies, the frequency response of the error signal goes down, explaining the observed behavior in the spectra in Fig. 1d in the main text. Because the sampling noise in the frequency estimation affects the qubit frequency through the feedback, the spectral density of the error signal does not exactly match the spectral density of the real qubit frequency.

We now analyze the impact of the feedback directly on the noise spectral density of the qubit frequency. We first calculate the transfer function $X_{\mathrm{f}}(z) = \frac{F(z)}{D(z)-V(z)-\tilde{F}(z)}$ of the qubit frequency $f[n]$ right after a feedback cycle when

$f[n] = p[n] + \tilde{f}[n]$. Using the result of Eq. (S10) we get

$$\begin{aligned} X_{\mathrm{f}}(z) &= \frac{\tilde{F}(z)}{D(z) - V(z) - \tilde{F}(z)} + X_{\mathrm{p}}(z) \\ \implies X_{\mathrm{f}}(z) &= \begin{cases} X_{\mathrm{p}}(z) - 1, & \text{for}\, V(z) = 0 \,\text{and}\, D(z) = 0 \\ X_{\mathrm{p}}(z), & \text{for}\, \tilde{F}(z) = 0 \,\text{and}\, D(z) = 0. \end{cases} \end{aligned} \tag{S14}$$

The squared magnitude of the frequency responses of these transfer functions, $|H_{\mathrm{f}}^{F(\tilde{z})=0}(f)|^2$ and $|H_{\mathrm{f}}^{V(\tilde{z})=0}(f)|^2$, are plotted in Fig. S3c. The spectral density of the qubit frequency fluctuations is then $S_{\mathrm{ff}}(f) = |H_{\mathrm{f}}^{\tilde{F}(z)=0}(f)|^2 S_{\mathrm{vv}}(f) + |H_{\mathrm{f}}^{\tilde{V}(z)=0}(f)|^2 S_{\tilde{\mathrm{f}}\tilde{\mathrm{f}}}(f)$, assuming we can independently sum the contributions from the sampling noise and the qubit frequency fluctuations, shown in Fig. S3d for the same parameters as used in Fig. 1d in the main text. Quite remarkably, this analysis very closely reproduces the simulation of the qubit frequency fluctuation spectral density shown with red in Fig. 1d.

D. Spectral density of flux noise

The measured coefficients $k_{\mathrm{E/R}}$ can be connected to the spectral density of $1/f$ flux noise, $S_{\Phi\Phi}(f) = A_\Phi/|2\pi f|$, as

$$\sqrt{A_\Phi} = \frac{k_{\mathrm{E/R}}}{2\pi\sqrt{\eta_{\mathrm{E/R}}}}, \tag{S15}$$

where the labels E or R refer either to echo or Ramsey sequence, and $\eta_{\mathrm{E/R}}$ is a scaling parameter which depends on the bandwidth of the noise to which the used pulse sequence is sensitive, $\eta_{\mathrm{E}} = \ln 2$, and $\eta_{\mathrm{R}} = \ln \frac{f_{\mathrm{u}}}{2\pi f_{\mathrm{l}}} \approx 13$ [29]. Here, we take the upper cutoff frequency $f_{\mathrm{u}} = 100\,\mathrm{kHz} \sim 1/T_2$ and the lower cutoff frequency $f_{\mathrm{l}} = 40\,\mathrm{mHz}$ corresponding to the inverse of the total duration of the Ramsey experiment. By substituting the measured values of k to the above formula, we infer $\sqrt{A_\phi} = 2.8\,\mu\Phi_0$ without the feedback. With the feedback activated, the flux noise spectral density no longer follows $1/f$ law due to the suppression of low frequency noise, but for comparison we can still calculate an effective flux noise amplitude using Eq. (S15), which yields $\sqrt{A_\phi} = 2.4\,\mu\Phi_0$. When measured using the echo sequence we get $\sqrt{A_\phi} = 3.3\,\mu\Phi_0$. Ideally, the flux noise amplitude inferred from the Ramsey experiment without the feedback should yield the same value as the flux noise amplitude calculated from the echo experiment, but we attribute the discrepancy to the deviation of the noise spectrum from the ideal $1/f$ spectrum, as observed earlier in Fig. 1b. The measured flux noise amplitude is consistent with the earlier systematic study we performed for flux noise amplitudes of SQUID loops of different sizes [11].

E. Analysis on fundamental limitations of the feedback implementation

Here we analyze fundamental limitations on the attainable improvements to the coherence time using the feedback implementation studied in this work. According to Eq. (5), the coherence time depends on the noise spectral density $S_{f_{\mathrm{q}},f_{\mathrm{q}}}(f)$ and the cutoff frequency f_0. In order to calculate the highest attainable coherence time, we need to find the feedback parameters that minimize the noise spectral density in the relevant frequency bandwidth. These parameters are the Ramsey delay τ, number of probing sequences N, and the gain of the feedback loop G. The optimal values of these parameters depend on the shape of the noise spectral density function, and we therefore use the spectral density shown in Fig. 1d as an example. For now we also assume $N = 1$ and $G = 1$ for simplicity. Fig. S4a shows a simulation where we sweep the Ramsey delay τ and calculate the resulting noise spectral density by assuming that feedback is fully efficient within its bandwidth, and add the statistical sampling noise as given in Eq. (4). We then evaluate the coherence time from Eq. (5). Without feedback the coherence time is independent of the feedback parameters (solid blue line). Under the made assumptions, the simulation shows that the feedback can improve the coherence time of the qubit by a factor of 4 (dashed orange line) at $\tau \approx 20\,\mu\mathrm{s}$. We stress that the factor significantly depends on the lower cutoff frequency f_0, as demonstrated in Fig. 2b, and is ultimately dictated by the total duration of the experiment. Here we have taken $f_0 = 13.5\,\mathrm{mHz}$.

The above simulation does not take into account that dephasing also reduces frequency estimation efficiency due to reduced visibility in the Ramsey experiment. To address that, we rewrite Eq. (S2) to include decoherence

$$p_1 = \frac{1}{2} + \frac{1}{2}\chi(\tau)\cos(2\pi\delta_{\mathrm{q}}\tau - \phi_{\mathrm{m}}), \tag{S16}$$

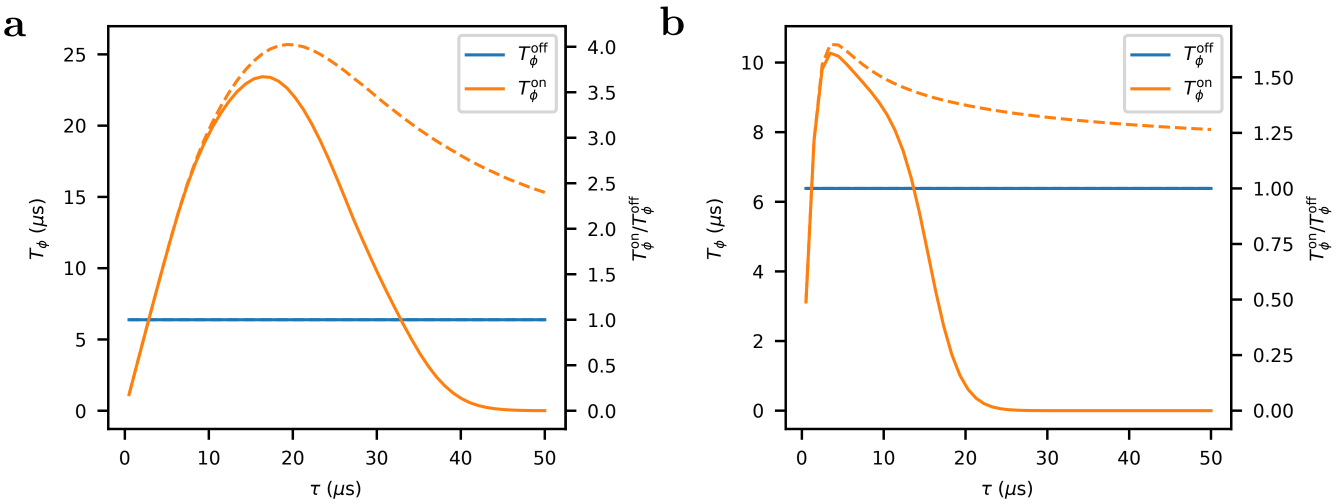

FIG. S4. The optimal performance of the feedback. a) Performance of the feedback is simulated with $G = 1$ and $N = 1$. Solid blue line shows the qubit coherence without feedback ($T_\phi^{\rm off}$), whereas the dashed orange line shows the qubit coherence with the feedback activated ($T_\phi^{\rm on}$). Solid orange line shows the coherence time when the impact of decoherence on the qubit frequency estimation is taken into account. The right axis shows the relative impact of the feedback. b) As in a, but with $N = 20$.

and following the steps in Section C derive the statistical sampling noise in the presence of decoherence as

$$S_{\rm est}(f) = \begin{cases} \frac{T}{2\pi^2\tau^2\chi(\tau)^2}, & 0 \le f \le \frac{1}{2NT}, \\ 0, & \text{otherwise.} \end{cases} \tag{S17}$$

The dephasing envelope $\chi(\tau)$ during the frequency estimation is determined by the noise spectral density $S_{f_{\rm q},f_{\rm q}}(f)$ and a cutoff $f_0^{\rm fe} = \frac{1}{N(\tau + t^{\rm overhead})}$, where we take $t^{\rm overhead} = 0$. With this, we can make a more realistic estimate of the optimal performance of the feedback, shown with solid orange line in Fig. S4a. The impact of decoherence on the optimal performance is still quite modest as $T_\phi^{\rm on}/T_\phi^{\rm off}$ drops from 4.0 to 3.7.

In the actual experiment we used significantly smaller values of τ due to the requirement that the changes in the qubit frequency between each steps of the feedback produce a unique response from a Ramsey experiment. Due to occasional rapid jumps in the qubit frequency - probably caused by parasitic two-level fluctuators - we noticed that $\tau < 5\,\mu$s was required to make sure that the qubit frequency stays locked to the correct frequency. This problem can be overcome by employing frequency estimation algorithm using several values of τ to increase the amplitude bandwidth while maintaining the sensitivity required to accurately cancel the noise, and remains a topic for future research.

Fig. S4a shows that feedback efficiency quickly drops for $\tau < 15\,\mu$s due to increasing sampling noise. To counteract that, we use $N = 20$ at the cost of reduced frequency bandwidth. Fig. 4b shows the result of the simulation. The feedback can now be operated with a significantly smaller $\tau = 3.5\,\mu$s at the cost of overall reduction in the efficiency, down to $T_\phi^{\rm on}/T_\phi^{\rm off} \approx 1.6$. In the experiment we can further tune the optimal operation point by changing the gain of the feedback loop G to tune the frequency bandwidth as we adjust τ.

F. Coherence limit in randomized benchmarking

The limit imposed on the gate fidelity by decoherence has been studied for example in [32, 39]. The error probability per gate due to decoherence depends on the gate time and the coherence times as

$$\epsilon = \frac{t_{\rm gate}}{3T_1} + \frac{t_{\rm gate}}{3T_{\phi 1}} + \frac{1}{3}\left(\frac{t_{\rm gate}}{T_{\phi 2}}\right)^2, \tag{S18}$$

where T_1 is the energy-relaxation rate of the qubit, $T_{\phi 1}$ is the exponential part of the pure dephasing time, and $T_{\phi 2}$ is the Gaussian part of the pure dephasing time. In the experiment, we have $t_{\rm gate} = 40\,$ns, which consists of 30 ns

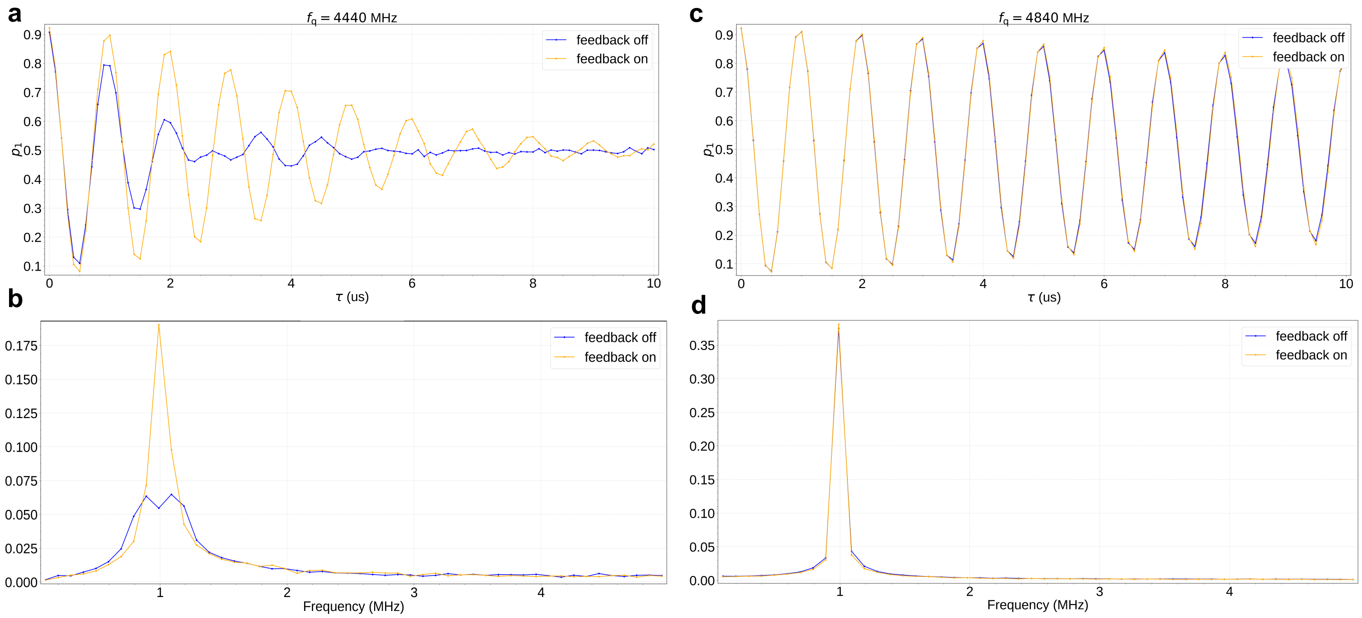

FIG. S5. Mitigating impact of TLSs using feedback. a) Ramsey oscillations at $f_{\mathrm{q}} = 4.440\,\mathrm{GHz}$ show beating behavior (blue line) which is mitigated by applying the feedback (orange line). b) Fourier transform of the Ramsey oscillations shown in panel a). c) A reference Ramsey oscillation measurement without beating, measured at the qubit flux sweet spot, $f_{\mathrm{q}} = 4.840\,\mathrm{GHz}$. d) The corresponding Fourier transform.

long cosine-shaped envelope and 10 ns delay between the pulses. The T_1 times of our qubit were between 26 µs and 40 µs, depending on the bias point used in the experiment. Using Eq. (S18), we calculate ϵ at all the 11 bias points used in the experiment, yielding values in the range of 3×10^{-4} to 5×10^{-4}, which are shown with black dots in Fig. 4c in the main text. The gate errors due to the Gaussian dephasing $\frac{1}{3}\left(\frac{t_{\mathrm{gate}}}{T_{\phi 2}}\right)^2 < 0.33 \times 10^{-4}$ contribute to the total error rate significantly less than the energy-relaxation rate, even at the most sensitive bias point where $T_{\phi 2} \approx 4\,\mu\mathrm{s}$. Feedback improved the dephasing time to $T_{\phi 2} \approx 5\,\mu\mathrm{s}$, which according to the estimate would reduce the Gaussian dephasing induced error rate to $\sim 0.2 \times 10^{-4}$. However, the measured decrease in gate errors was significantly higher, from $(8.5 \pm 2.1) \times 10^{-4}$ to $(5.9 \pm 0.7) \times 10^{-4}$. We attribute the higher efficiency of the feedback to its ability to correct slow frequency fluctuations of the qubit, not captured by a single T_2 experiment.

G. Additional analysis on mitigating the impact of two-level fluctuators using feedback

At certain qubit operating frequencies we noticed that in a Ramsey experiment there are two super-imposed oscillation frequencies, see Fig. S5a for a Ramsey trace measured at $f_{\mathrm{q}} = 4.440\,\mathrm{GHz}$ and Fig. S5b for the Fourier transform of the oscillations. One typical source for such beating behavior are parasitic two-level systems (TLS) [25]. When we turn on the feedback, the beating behavior vanishes, supporting our conclusion that feedback can suppresses qubit frequency fluctuations due to TLSs, thereby increasing the gate fidelity.
